# Switching Monopolar Radiofrequency Ablation Using a Separable Cluster Electrode in Patients with Hepatocellular Carcinoma: A Prospective Study

**DOI:** 10.1371/journal.pone.0161980

**Published:** 2016-08-30

**Authors:** Jin Woo Choi, Jeong Min Lee, Dong Ho Lee, Jeong-Hee Yoon, Kyung-Suk Suh, Jung-Hwan Yoon, Yoon Jun Kim, Jeong-Hoon Lee, Su Jong Yu, Joon Koo Han

**Affiliations:** 1 Department of Radiology, Seoul National University Hospital, Seoul, Korea; 2 Department of Surgery, Seoul National University Hospital, Seoul, Korea; 3 Department of Internal Medicine, Seoul National University Hospital, Seoul, Korea; Taipei Veterans General Hospital, TAIWAN

## Abstract

**Objective:**

This study was conducted to evaluate the outcomes of multi-channel switching RFA using a separable cluster electrode in patients with HCC.

**Methods:**

From November 2011 to July 2013, 79 patients with 98 HCCs < 5 cm were enrolled and treated with RFA using a multi-channel switching radiofrequency system and a separable cluster electrode under the guidance of a real-time fusion imaging system. The primary and secondary endpoints were the 3-year local tumor progression (LTP) rate and recurrence-free survival (RFS) rate, respectively. For post hoc analyses, LTP, RFS, and major complication rates were retrospectively compared with a historical control group treated with RFA using the same radiofrequency system but with multiple internally-cooled electrodes.

**Results:**

The technique success rate of the 98 tumors was 100%. Cumulative 1-year, 2-year, and 3-year LTP rates were 3.4%, 6.9%, and 12.4%, respectively. For patient-level data, cumulative 1-year, 2-year, and 3-year RFS rates were 83.9%, 68.6%, and 45.4%, respectively. On post hoc analyses, none of the baseline characteristics showed a significant difference between the separable cluster electrode and multiple internally-cooled electrodes group. Cumulative LTP and RFS rates of the two groups also showed no significant difference (*p* = 0.401 and *p* = 0.881, respectively). Finally, major complication rates of the separable cluster electrode group (5.0%, 4/79) and multiple internally-cooled electrodes group (5.9%, 4/74) were also comparable (*p* = 1.000).

**Conclusion:**

Switching monopolar RFA using a separable cluster electrode is a feasible and efficient technique for the treatment of HCCs smaller than 5 cm, providing comparable local tumor control to multiple internally-cooled electrodes.

**Trial Registration:**

ClinicalTrials.gov NCT02745483

## Introduction

Radiofrequency ablation (RFA) has been widely utilized as an effective treatment option for hepatocellular carcinoma (HCC) as well as diverse liver metastases [1–7]. Compared to surgical resection, RFA is less invasive, has less morbidity and requires shorter periods of hospitalization, while providing comparable outcomes [8]. Moreover, RFA has been reported to provide better cost effectiveness than surgical resection, especially in patients with single, small HCCs ≤ 2 cm [9–12]. A previous meta-analysis study [12] reported that RF ablation performed with conventional overlapping RFA for early HCC using various kinds of electrodes provided a pooled estimate of 3-year survival of 74.8%, compared to 79.8% observed for surgical resection. However, until now, RFA has been limited in achieving local tumor control for tumors larger than 3 cm compared with surgical resection due to its difficulty in creating a sufficiently large ablation volume including the target tumor and a 5−10 mm safety margin (3-year disease-free survival: 45.8% for surgery vs. 29.9% for RFA) [12]. Therefore, various strategies have been recently employed to create a sufficient ablation zone, including the use of multi-tined electrodes (RITA Medical Systems, Mountain View, CA) to increase the active surface area [13, 14], clustered internally-cooled electrodes (Covidien, Burlington, Mass) to diminish charring [15, 16], perfusion electrodes to promote ionic availability [17], switching monopolar or multipolar controllers to provide a synergy of multiple applicators [18–20], and high-power generators to increase power which would help overcome impedance [21].

In this context, multiple-electrode RFA using a switching radiofrequency system has emerged as one of the most promising techniques, with reports thus far describing competent short-term and mid-term results, albeit at an increased cost owing to the use of multiple electrodes [20, 22]. In addition, although the use of clustered electrodes has been shown to create a large ablation zone [23], technical problems still remain including the difficulty of converging the three needles in an area < 5 mm, difficulty in placing the electrodes in patients with narrow intercostal spaces or in those with a severely fibrotic liver exhibiting increased resistance to the electrode. Thus, in order to overcome the potential technical problems of clustered internally-cooled electrodes and to detour the problem of the increased cost of the multiple electrode approach, a separable cluster electrode consisting of one adapter and three active applicators which can be incorporated into a single handle as in usual cluster electrodes with 0.5 cm inter-tine distances, or separated into three independent applicators has recently been made commercially available [24]. Indeed, in a recent *in vivo* porcine study [24] switching monopolar RFA using a separable cluster electrode, with which the inter-tine distances can be manipulated by the operator, was shown to be more efficient in creating a large ablation zone than conventional cluster electrodes. Yet, although this separable cluster electrode has demonstrated promising results in a pre-clinical study [25, 26], the clinical feasibility and efficacy of this novel device has yet to be demonstrated in human studies.

Therefore, we performed a prospective clinical trial to evaluate the clinical feasibility and outcomes of switching monopolar RFA using a separable cluster electrode in patients with HCC. In addition, we compared the therapeutic outcomes and safety of the study patients with those of a historical control group using switching monopolar RFA with multiple internally-cooled electrode in patients with small- and medium- sized HCCs (< 5 cm).

## Materials and Methods

### Study Design

The institutional review board of Seoul National University Hospital approved this study, and all patients agreed to their registration with written, informed consent prior to the procedures. A single-arm, prospective study was conducted at a single medical center in order to evaluate the clinical feasibility and outcomes of RFA using a separable cluster electrode (Octopus^®^, STARmed; Goyang-si, Gyunggi-do, Republic of Korea) and a multi-channel switching radiofrequency system (STARmed) for the treatment of liver malignancies (S1 Protocol and S1 TREND Checklist). After completion of the original study (NCT02683538) which addressed clinical feasibility, this study was additionally registered at *ClinicalTrials*.*gov* (NCT02745483) and conducted to evaluate long-term survival with more statistical power (i.e. longer follow-up periods) and to compare the results with a historical control group. Both studies were initially registered at an institutional clinical study database (cris.snuh.org) and unpublished to secure the novelty of this new RFA device, and then were declared at *ClinicalTrials*.*gov* for publication after the study completion. This study was reported according to Consolidated Standards of Reporting Trials recommendations (www.consort-statement.org) and Transparent Reporting of Evaluations with Nonrandomized Designs (www.cdc.gov/trendstatement).

The primary endpoint was the cumulative 3-year local tumor progression (LTP) rate after RFA. The secondary endpoint was the cumulative recurrence-free survival (RFS) rates after RFA. Tertiary endpoints included the technique success rate and major complication rate [27]. In addition, as a post hoc study, these aforementioned estimates were retrospectively compared with a historical control group treated with RFA using multiple internally-cooled electrodes and the same radiofrequency system. The authors confirm that all ongoing and related trials for this intervention are registered.

### Patients

**Fig 1:**
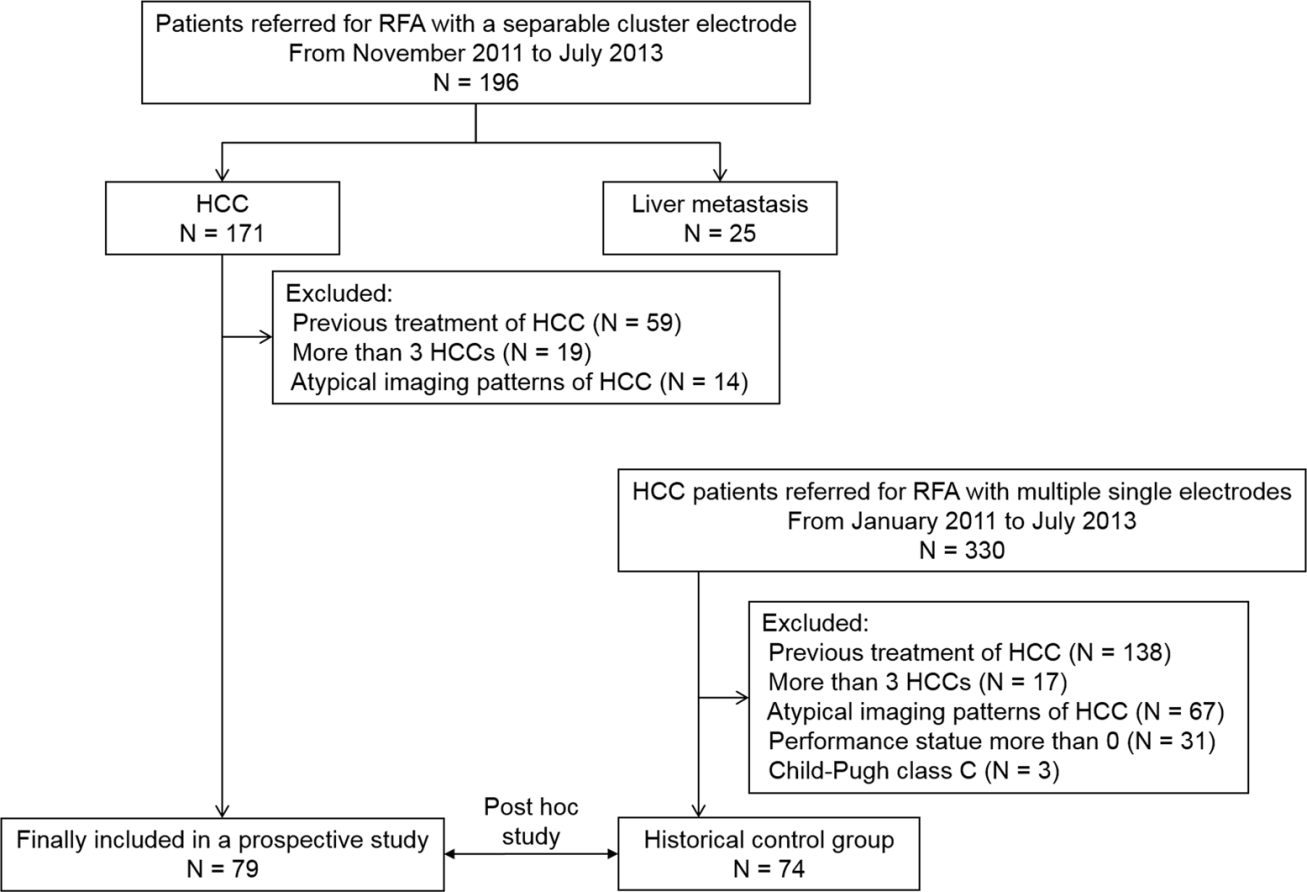
Flowchart of the study population enrollment.

Eligible patients were extracted from the original prospective study (n = 196) to evaluate the clinical feasibility of a separable cluster electrode for hepatic malignancies. The inclusion criteria of this study were as follows: 1) HCCs diagnosed on image-guided biopsy or on dynamic computed tomography (CT) or magnetic resonance (MR) imaging [1, 2]; 2) 1−3 HCCs ≤ 5 cm in the liver; 3) no direct contact with or invasion into hepatic hilar structures or the inferior vena cava; 4) treatment-naive patients; and 5) patients with an Eastern Cooperative Oncology Group performance status of 0. Image-guided biopsy was conducted for patients whose imaging features were not characteristic [1, 2]. We excluded patients per the following exclusion criteria: 1) laboratory evidence of coagulopathy, i.e., a platelet count < 50,000/μL or an international normalized ratio of prothrombin time > 1.5; 2) compromised liver function according to Child-Pugh class C; 3) radiological evidence of tumor invasion into the portal vein or hepatic vein; 4) radiologic evidence of extrahepatic spread of the HCC, and 5) severe cardiac or pulmonary diseases. The imaging diagnosis of HCC was based on the American Association for the Study of Liver Disease practice guidelines [1]. Using these criteria, from November 2011 to July 2013, a total of 79 patients with 98 HCCs were enrolled in this study (Fig 1). Sixty-three patients (79.7%, 63/79) had single HCCs, and the remaining 16 patients (20.3%, 16/79) had multiple tumors: 13 patients with 2 HCCs, and 3 patients with 3 HCCs. The diameter of the 98 nodules was 0.6 to 4.1 cm (mean ± standard deviation, 1.9 cm ± 0.7). The baseline characteristics of the study patients are summarized in Table 1.

**Table 1:** Baseline characteristics of the study population.

Variable	Electrode used with the switching RFA system		*p*-value
	Separable cluster electrode (79 patients with 98 HCCs)	Multiple IC electrodes (74 patients with 88 HCCs)	
**Sex**			.913
Male	53 (67.1)	50 (67.6)	
Female	26 (32.9)	24 (32.4)	
**Age**, years^†^	61.7 ± 9.1	62.4 ± 8.4	.617
**Child-Pugh class**			.747
A	73 (92.4)	70 (94.6)	
B	6 (7.6)	4 (5.4)	
**Tumor size**, cm^†^	1.9 ± 0.7	1.8 ± 0.7	.253
< 2 cm	44 (44.9)	62 (70.5)	
2−5 cm	54 (55.1)	26 (29.5)	
**Etiology of HCC**			.538
Viral	68 (86.1)	60 (81.1)	
Non-viral	11 (13.9)	14 (18.9)	
**Number of HCC**			.997
Single	63 (79.7)	60 (81.1)	
Multiple	16 (20.3)	14 (18.9)	
**Perivascular location**			.432
Yes	25 (25.5)	28 (31.8)	
No	73 (74.5)	60 (68.2)	
**Subcapsular location**			.106
Yes	40 (40.8)	25 (28.4)	
No	58 (59.2)	63 (71.6)	
**Alpha feto-protein**, ng/mL^†^	61.7 ± 9.7	153.5 ± 454.1	.156
**Diagnosis of HCC**			.160
Biopsy	8 (8.2)	14 (15.9)	
Imaging	90 (91.8)	74 (84.1)	

Note.− Numbers in parentheses are percentages. IC = internally cooled

^†^ Data are mean ± standard deviation.

### CT Image Acquisition for Image Fusion

All patients also underwent monophasic late arterial phase CT scans on the same day as RFA, using a 128-row detector scanner (Discovery CT750HD; GE Healthcare, Waukesha, WI, USA) at a low kilo-voltage peak (80 kVp) setting so as to reduce the radiation dose while increasing the contrast-to-noise ratio between the index tumor and the liver background [28]. Intravenous administration of a nonionic iodinated contrast agent (1 mL/kg of iopromide, Ultravist 370; Bayer Healthcare, Berlin, Germany) at a rate of 2–4 mL/sec was used to visualize the index tumor in all patients, followed by a saline flush of approximately 30- to 40-mL [29, 30]. A bolus-tracking program (Smart-Prep; GE Healthcare) was used to commence diagnostic scans after contrast media injection, which was started 20 seconds after reaching the 100 HU contrast enhancement threshold [29] at the mid-phase of the breath–hold, mimicking shallow breathing under conscious sedation, with three pairs of sterile, passive, fiducial markers placed on the skin of the lower chest [31]. Images were then reconstructed using a slice thickness of 2.5 mm with a 1.25 mm overlap using adaptive iterative reconstruction (ASIR; GE Healthcare) for fusion with intra-procedural ultrasonography (US) images.

### RFA

#### Ablation Protocol

All procedures were conducted on an inpatient basis with curative intent by two of three experienced radiologists (J.M.L., J.H.Y., and D.H.L with 16, 7 and 7 years of experience in US-guided interventional procedures including RFA). The goal of RFA was to achieve complete ablation of both the visible tumor and a 0.5- to 1-cm-wide ablation margin in the normal liver parenchyma surrounding the tumor [27, 32]. During the procedure, patients were put under conscious sedation, and patients’ blood pressure, pulse rate, electrocardiography, and oxygen saturation levels were continuously monitored. Local anesthesia was performed by the operator using a subcutaneous injection of 5−15 mL of 1% lidocaine (Dai Han Pharm, Seoul, Korea).

**Fig 2:**
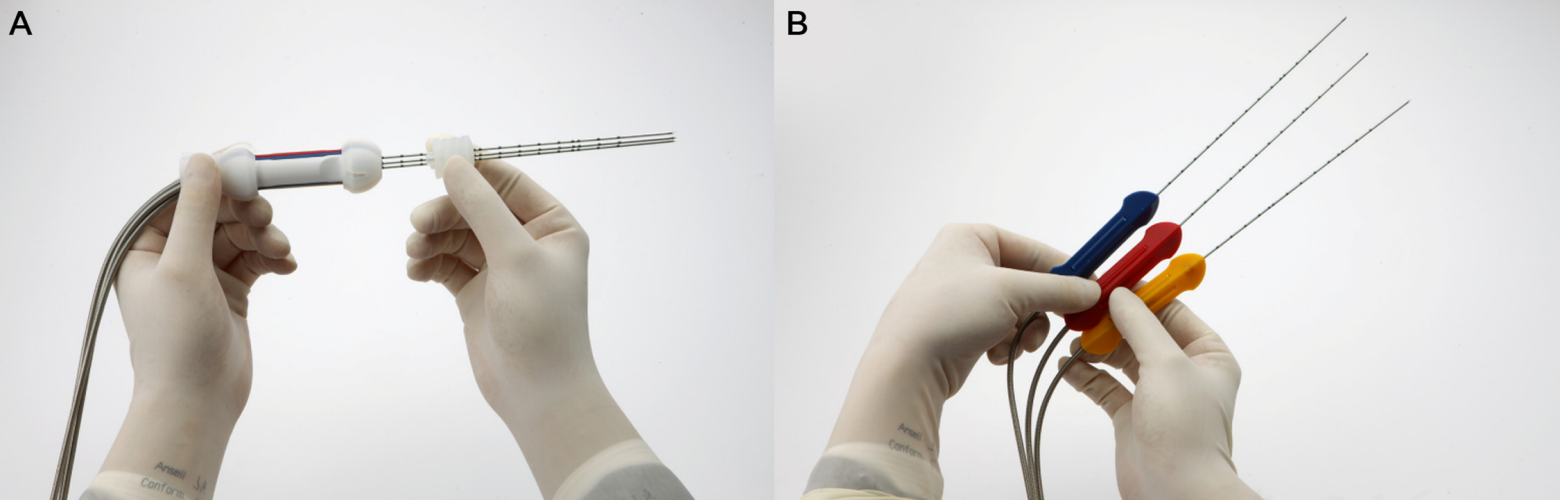
Diagram of the “no-tumor-touch” technique to ablate a 1.5 cm sized HCC.

One cycle of ablation was performed for approximately 10 minutes in tumors < 2.5 cm and for 18 minutes in tumors > 2.5 cm [24]. When tumors < 2 cm were completely covered in echogenic bubbles with a sufficient ablation margin within 8 minutes, we terminated the procedure at that time point. If an optimal ablation margin was not achieved after the first cycle of ablation, additional cycle(s) of RFA were done followed by repositioning of the electrode(s), as appropriate [33]. Depending on the tumor size, shape, location, and the presence of large, adjacent vessels, the operator determined and adjusted the length of the active tips among three options, 2.5 cm, 3.0 cm, and 4.0 cm [20]. In general, if the tumor was smaller than 1.5 cm in its longest axis, electrodes with 2.5 cm active tips were used using the “no-tumor-touch” technique [34]. We used an ablation technique, in which the electrodes were inserted not into the tumor but into the perimeter of the index tumor with a 2 cm inter-electrode distance, in order to avoid aggressive intrasegmental recurrence of HCC or possible track seeding after RFA [35] (Fig 2). If the tumor was 1.5−3.0 cm in its longest axis, electrodes with 3.0 cm active tips were used. Finally, if the tumor was larger than 3.0 cm, electrodes with 4.0 cm active tips were used. Grounding was achieved by attaching four, dispersive pads to the patients’ thighs. Artificial ascites (5% dextrose solution), used to ablate subcapsular tumors or tumors located near the diaphragm, were aspirated as much as possible after the procedures [36]. At the end of the RFA procedure, the tracts where each applicator passed were ablated by maintaining the active tips at 90°C while retracting the electrodes in order to prevent bleeding and tumor seeding.

#### Fusion Imaging-guidance

To determine the location of the index tumor and the relationship of the tumors with adjacent intrahepatic vessels as well as optimal positions for the electrodes, a real time fusion imaging technique between pre-procedural CT imaging and intra-procedural US imaging was utilized as previously described, [31, 37]. Fusion imaging was achieved using an electromagnetic navigation system (PercuNAV; Philips Healthcare, Best, Netherlands) which consisted of a magnetic field generator placed near the patient and a sensor attached to an US transducer used for spatial tracking of the US probe. Pre-procedural CT images were transferred to the US navigation system (IU 22, and PercuNAV, Philips) located in the procedure room and automatic registration between the CT images and real-time US images was performed using three pairs of sterile, passive, fiducial markers placed on the skin of the lower chest, according to the vendor’s recommendations. If automatic registration failed to provide high quality registration, plane registration between the CT images and US images was conducted using an image plane, showing anatomical landmarks such as the portal vein, inferior vena cava, and hepatic vein [31]. Afterwards, point registration was performed to correct any minor registration errors, using focal hepatic lesions such as cysts and calcification around the index tumor. The registration was finally checked by confirming that the center of the index tumor appeared in the same plane on real-time US and CT images. The image registration process took approximately one to six minutes. After image fusion, real-time B-mode ultrasound and fused CT/US images with a 3D virtual ablation sphere including the index tumor and a 5 mm safety margin were displayed simultaneously on a split-screen display. During RFA, the precise location of the electrodes and the relationship between the 3D virtual ablation sphere including the index tumor and ablation zone with echogenic bubbles were carefully monitored using real-time CT/US fusion imaging.

During registration, patients were put under conscious sedation. If artificial ascites instillation was required to safely ablate subcapsular HCCs, image fusion was performed following the instillation. Based on the fusion-images, the operator decided the route and position of each electrode. After placing the separable cluster electrode, radiofrequency energy was applied for approximately 8−18 minutes using an impedance-switching algorithm until the ablative zone was considered to cover the entire tumor [19, 24, 38]. However, when the area of echogenic bubble clouds was not considered to cover the virtual margin of the tumor including at least a 5 mm safety zone on fusion images, radiofrequency energy was additionally applied after repositioning one or two electrodes to different sites of the tumors.

#### Switching radiofrequency system with a Separable Cluster Electrode

**Fig 3:**
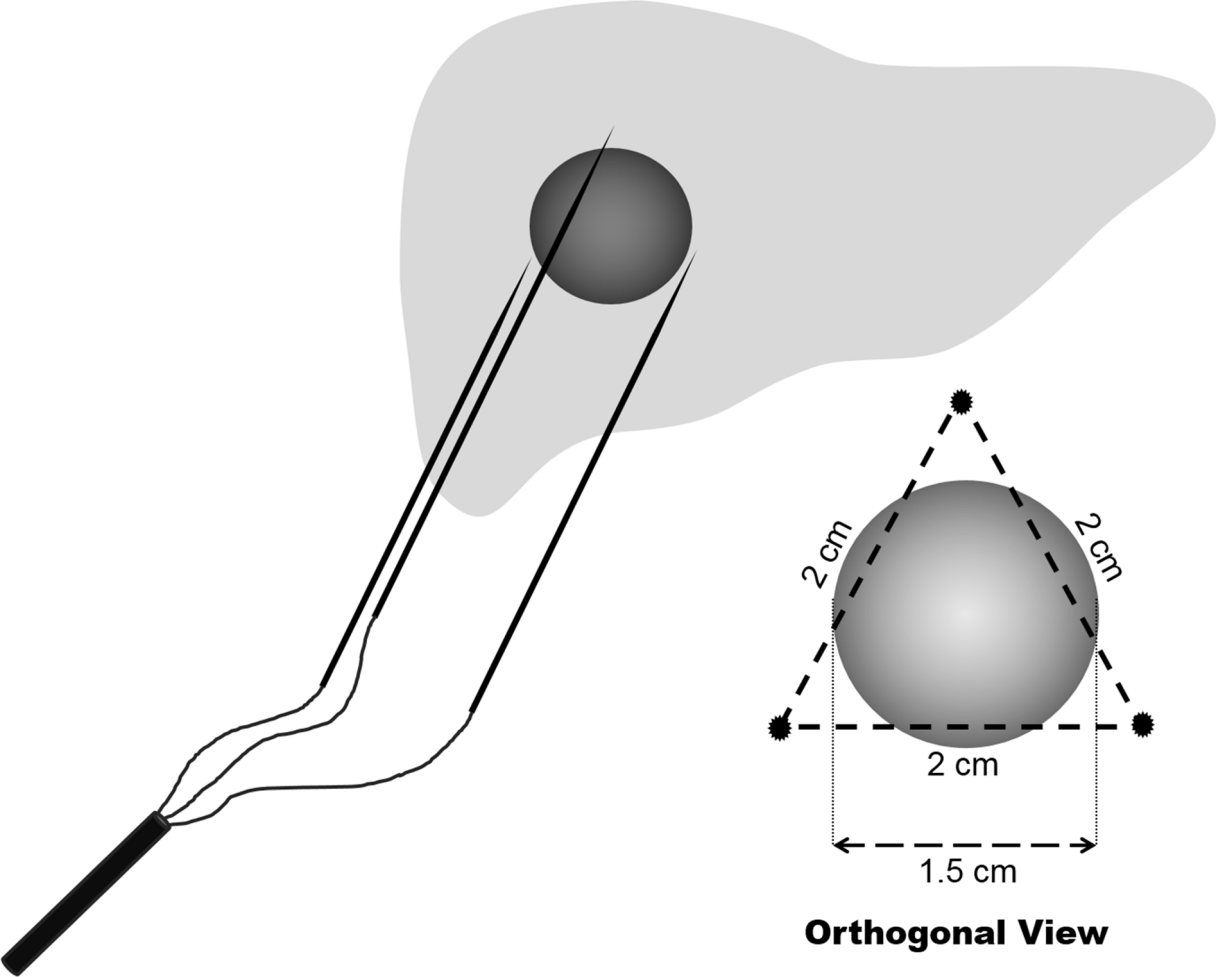
Photographs of a separable cluster electrode (Octopus^®^, STARmed) composed of three internally-cooled electrodes that can be incorporated as (A) one cluster electrode with a large shaft, or separated as (B) three individual applicators with small handles.

A separable cluster electrode (Octopus^®^; STARmed), in which the inter-tine distances can be manipulated by the operator, was used in all patients (Fig 3). In our study, a 200-watt, multichannel, radiofrequency system (Viva RF System; STARmed) with three independently adjustable generators were used, allowing independent control of radiofrequency energy delivery to each applicator. Therefore, the separable cluster electrode was used as three, internally-cooled, single electrodes, thereby providing three ablation points with the same generator (Table 2). For tissue heating, radiofrequency energy was delivered, alternating among the multiple electrodes in the single switching monopolar mode [20]. In our switching radiofrequency system, the active electrode was switched when the impedance increased 50 Ω above baseline or when the ablation time passed 30 seconds [24]. During energy delivery, chilled normal saline was circulated in the lumen of the electrode to keep the active tip temperature at 20−25°C. The detailed algorithm of energy application was followed as per the manufacturer’s instructions.

**Table 2:** Differences between separable cluster electrodes and other electrodes.

	Separable cluster electrode	Conventional cluster electrode	Single, internally-cooled electrode
The number of active tips	3	3	1
Inter-tine distance	Adjustable (separated state) or fixed (incorporate state)	Fixed	Adjustable
The number of electrodes required for a large tumor	1 to 2	1 (less effective)	3 or more
Multiple overlapping technique	Available	Available (limited for small tumors)	Available
Occupancy of the intercostal space	Small	Large	Small

### Procedure Evaluation

Following recovery from conscious sedation after RFA (10−20 minutes after RFA), arterial- and portal-phase imaging with multiplanar reconstruction (axial, coronal, and sagittal) was performed on the same CT scanner using the low-tube-voltage CT protocol of our institution and the automatic tube-current modulation technique (100 kVp; a noise index of 10.7 HU at 5-mm slice collimations; tube current, variable; detector configuration, 64 × 0.625 mm; beam collimation, 40 mm; and rotation time, 0.5 s). Contrast medium (1.35 mL/kg of Ultravist 370; Bayer Healthcare) was administered intravenously at a rate of 2.0 to 4.0 mL/s using a power injector (Multilevel CT; Medrad, Indianola, PA), followed by a saline flush of approximately 30 to 40 mL. The arterial phase was obtained 19 seconds after the attenuation of the descending aorta reached 100 HU as measured using the bolus tracking method, and the portal venous phase was obtained approximately 70 seconds after beginning contrast media administration. These images were obtained in order to determine technical success, ablation volume, and to detect procedure-related complications.

The technique success of RFAs was defined as complete coverage of the index tumor with a low attenuating area on the portal phase, and an extended ablation zone beyond the tumor border on immediate post-RFA CT, according to the standardized terminology of the International Working Group on Image-Guided Tumor Ablation [27]. If technical success was not achieved, additional RFA was conducted immediately after the CT scan, under the guidance of US and post-RFA CT fusion imaging.

Regarding the ablation volume, we assumed that the ablation zone was spherical and thus used the following formula to calculate the ablation volume:

$$Ablation\;volume=\frac{\pi \times Dmax\times Dmin\times Dvert}{6}$$,

where Dmax and Dmin are the longest and shortest diameters of the ablation zone seen on the axial image showing a maximum area, and Dvert is the longest vertical (superoinferior) diameter seen on the coronal reconstructed image [19, 24]. All imaging analyses were conducted by an experienced radiologist blinded to the use of a separable cluster electrode.

The total procedure time was calculated as the time between the start of planning the US study before image fusion until the awakening of the patient from conscious sedation at the end of the procedure. Ablation time was considered as the period of time when energy was actively delivered via the electrodes.

### Complications

Major complications were defined as events increasing the level of care or lengthening the hospital stay [27]. If a patient died within 30 days after the RFA, it was regarded as a procedure-related death [27]. Post-ablation syndrome, which consists of transient and self-limiting symptoms of low-grade fever and general malaise, was also reported, but was not regarded as a major complication [27].

### Follow-up

All patients were followed up until July 2015, and the data were censored at the date of the last follow-up imaging after RFA. All patients underwent initial follow-up one month after the procedure, followed by regular follow-up every three months with quadriphasic (unenhanced, arterial, portal, and delayed phase) CT imaging to judge the technique efficacy, LTP, and HCC recurrence. Technique efficacy was determined based on whether complete ablation of the index tumor was achieved on one month follow-up CT images [27]. LTP was defined as when tumor foci appeared at the edge of the ablation zone [27]. All data were assessed by means of intention-to-treat analyses.

### Post hoc comparison with the multiple internally-cooled electrode group

To compare the RFA outcomes of separable cluster electrodes and multiple internally-cooled electrodes, a group of patients who underwent switching monopolar RFA with multiple internally-cooled electrodes and the same radiofrequency system (Viva RF System; STARmed) for HCC was collected retrospectively. Our institutional review board approved the additional, retrospective study, and permitted the waiver of informed consent.

From January 2011 to July 2013, a total of 361 patients with 619 HCCs, who were not prospectively enrolled in the study, were treated using RFA at our institution. Among them, switching monopolar RFA with singe electrodes was conducted for 330 patients. After applying the same inclusion and exclusion criteria as for the study group, 74 patients with 88 HCCs were selected for post hoc, retrospective comparison. The baseline characteristics of the patients are summarized in Table 1.

According to the routine protocol of our institution, these patients were treated using the same protocols of pre-procedural imaging acquisition, image guidance, and follow-up imaging and analyses. However, these patients received switching RFA with multiple internally-cooled electrodes, and the follow-up intervals were relatively irregular compared with the separable cluster electrode group.

### Statistical Analysis

Cumulative LTP rates and RFS rates at 1 year, 2 year, and 3 years were calculated using the Kaplan-Meier method. The time-to-LTP, at tumor-level data, was calculated as the length of time after RFA during the first LTP. If a patient died without LTP, time-to-LTP was censored at the date of death. RFS, at patient-level data, was defined as the length of time after RFA to death or the first recurrence of the HCC on follow-up imaging. Recurrence was classified as LTP with or without intrasegmental spread, intrahepatic distant recurrences (IDR) and extrahepatic spread [27].

For post hoc analyses, baseline characteristics of the separable cluster electrode group and multiple internally-cooled electrode group were compared using the chi-square test or Fisher’s exact test for categorical variables, and the independent t-test or Mann-Whitney test for numerical variables. Cumulative LTP and RFS rates of the two groups were compared using the log-rank test. To determine factors affecting LTP, RFS after RFA, baseline characteristics and the type of electrodes were evaluated using univariate Cox proportional hazard regression. Thereafter, multivariate analyses for significant factors were conducted using multivariate Cox proportional hazard regression (enter method). The proportional hazard assumption and goodness-of-fit of the model were verified using the log-minus-log plot and Cox-Snell residuals, respectively. Major complication rates of the two groups were compared using the Fisher’s exact test.

A *p-*value of less than .050 was considered to indicate a statistical significance. All statistical analyses were performed using commercial statistics software (MedCalc, version 15.8; MedCalc Software, Ostend, Belgium).

## Results

### Technical outcomes

**Fig 4:**
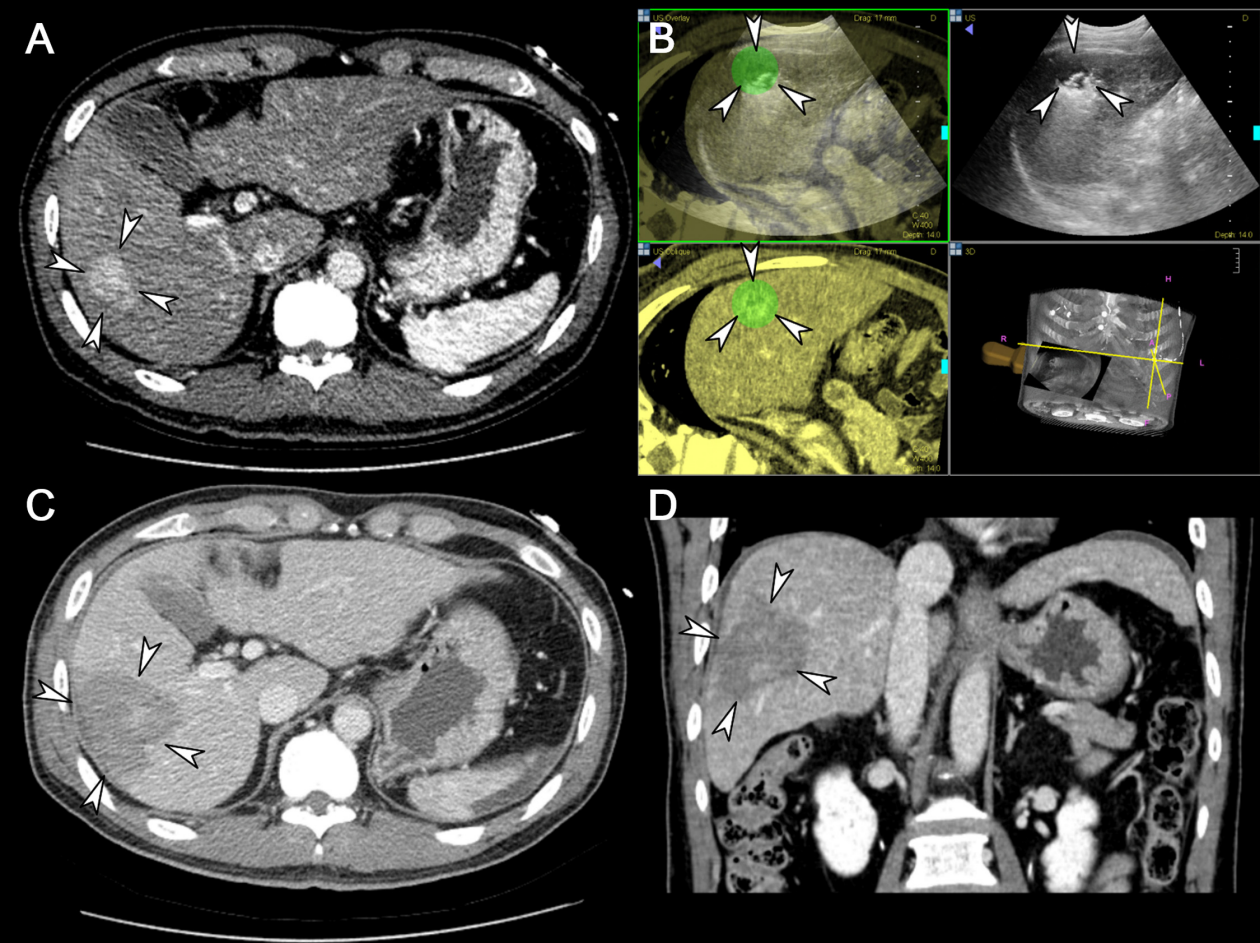
A representative case showing the usefulness of a separable cluster electrode in ablating a large volume at one time. (A) Axial CT image taken prior to RFA demonstrates a 3.4 cm sized, hypervascular lesion in the right lobe of the liver (arrowheads). (B) Intra-procedural US images fused with pre-procedural CT images guide the tumor (arrowheads) targeting and monitoring. (C) Axial CT image acquired immediately after RFA shows the ablation zone (arrowheads) sufficiently covering the index tumor, measured as 6.0 cm in long diameter, including the safety margin. (D) Coronal CT image reconstructed from the immediate post-procedural CT scan also depicts the ablation zone (arrowheads) measured as 5.9 cm in its coronal long axis.

The technique success rate of the 98 tumors was 100% (Fig 4). Total procedure times in patients with a single HCC (n = 63) and multiple HCCs (n = 16) were 50.3 ± 12.6 minutes (range, 30−95 minutes) and 58.1 ± 13.5 minutes (range, 40−90 minutes), respectively. The ablation time and volume per index tumor were 12.5 ± 5.5 minutes (range, 5−34 minutes) and 32.1 ± 18.1 cm^3^ (range, 4.9−86.3 cm^3^), respectively. An average of 64.6 ± 38.4 kJ of energy was applied (range, 8.8−245.7 kJ) to ablate the individual tumors. One patient with two HCCs did not undergo any follow-up study, and thus was excluded from further analyses. Technique efficacy was achieved in all patients except in one case of follow-up loss.

### Local tumor progression and recurrence free survival rates

With regard to the 98 HCCs, the cumulative 1-year, 2-year, and 3-year LTP rates were 3.4% (standard error [SE], 1.9%), 6.9% (SE, 2.7%), and 12.4% (SE, 4.7%), respectively. Among the ten index tumors ≥ 3.0 cm, no HCCs showed LTP after RFA during the follow-up period (range, 22.4−42.7 months). There was also an index tumor which developed aggressive intrahepatic recurrence of HCC after RFA.

At patient-level data, the cumulative 1-year, 2-year, and 3-year RFS rates were 83.9% (SE, 4.3%), 68.6% (SE, 5.4%), and 45.4% (SE, 7.5%), respectively. During the follow-up period, LTP, IDR, and extrahepatic spread occurred in six (7.6%, 6/79), 27 (34.2%, 27/79) and one (1.3%, 1/79) patient, respectively. Three patients (3.8%, 3/79) experienced both LTP and IDR.

### Complications

Four out of 79 patients (5.1%) experienced a major complication; pleural effusion requiring tube drainage (n = 2), non-fatal sepsis (n = 1), and peritoneal tumor seeding possibly owing to tumor spread through the electrode tract (n = 1). The patient with peritoneal tumor seeding was diagnosed four months after RFA, then managed with sorafenib for two months and through supportive care for one month, and finally was referred to a hospice. The remaining three patients were discharged after adequate management, without any adverse sequelae. There were no procedure-related mortalities. Post-ablation syndrome resulting in hospitalization longer than three days after RFA occurred in 11 patients (13.9%, 11/79), and five of them underwent multiple tumor ablations at a session.

### Post hoc comparison with the multiple internally-cooled electrode group

**Fig 5:**
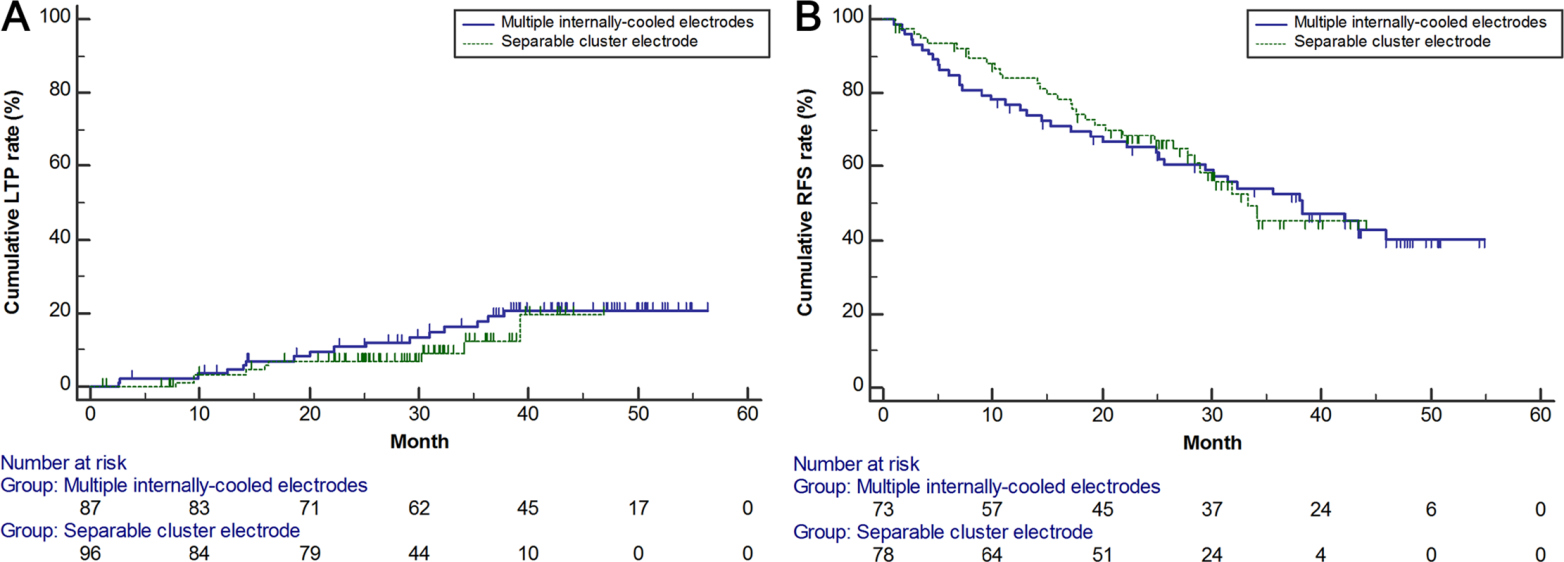
Kaplan-Meier curves showing cumulative (A) local tumor progression and (B) recurrence-free survival rates after switching RFA of HCC, in the separable cluster electrode group and multiple internally-cooled electrode groups (*p* = .401 and *p* = .881, respectively).

There were no significant differences between the baseline characteristics of the separable cluster electrode group and of the multiple internally-cooled electrode group (Table 1). The ablation time and volume of the multiple internally-cooled electrode group were 15.9 ± 6.7 minutes (range, 5−41 minutes) and 36.3 ± 21.4 cm^3^ (range, 4.2−112.1 cm^3^), respectively. They were not significantly different to those of the separable cluster electrode group (*p* = .055 and *p* = .149, respectively). The cumulative LTP rates (3.5%, 10.8%, and 17.8% at 1-year, 2-year, and 3-year, respectively) and RFS rates (76.7%, 65.1%, and 52.5% at 1-year, 2-year, and 3-year, respectively) of the multiple internally-cooled electrode group were also not significantly different than those of the separable cluster electrode group (*p* = .401 and *p* = .881, respectively) (Fig 5). In addition, according to Cox proportional hazard regression, LTP was not significantly related with the type of electrode or the baseline characteristics: the use of a separable cluster electrode did not significantly affect LTP (hazard ratio [HR], .701; 95% confidence interval [CI], .306 to 1.606). RFS was only significantly associated with Child-Pugh class (class B; HR, 8.912; 95% CI, 3.816 to 20.814; *p* < .001): the use of a separable cluster electrode did not significantly affect RFS (HR, .964; 95% CI, .596 to 1.559). The major complication rates of the separable cluster electrode group (5.0%, 4/79) and the electrode group (5.9%, 4/74) were not significantly different (*p* = 1.000).

## Discussion

This study demonstrated that switching monopolar RFA using a separable cluster electrode and a multi-channel radiofrequency system was clinically feasible, providing good mid-term LTP (12.4% at 3-year after RFA) and cumulative RFS (45.4% at 3-years after RFA) rates. In terms of therapeutic effectiveness, we found in our study that LTP (95% CI of HR, .306 to 1.606) and RFS (95% CI of HR, .596 to 1.559) rates of switching monopolar RFA using a separable cluster electrode were comparable with the historical control group who underwent RFA using multiple internally-cooled electrodes. Our study results were also in good agreement with a previous study using a multiple electrode switching system (11% local recurrence rate at 3-year after RFA) [20]. Furthermore, our study results on switching monopolar RFA using a separable cluster electrode were better than those reported in several other investigations [39–41]. With these results showing the comparative performances of separable cluster electrodes and multiple electrodes, we can suggest several potential advantages of the separable cluster electrode compared with the use of multiple internally-cooled electrodes. First, the separable cluster electrode provides versatile applications to operators, as it can be utilized with a switching monopolar system as well as a simultaneous monopolar system. Second, as the separable cluster electrode can be incorporated into a single handle as in usual cluster electrodes with 0.5 cm inter-tine distances, and that it can also be separated into three independent applicators, separable cluster electrodes can provide operators with more flexibility than clustered electrodes or multiple internally-cooled electrodes when treating a large tumor or multiple variable sized tumors. Last, a separable cluster electrode requires a single chiller per three radiofrequency applicators, which may be advantageous when the generator or spatial capacity is limited. Based on our study results, we believe that the separable cluster electrode is comparable with multiple internally-cooled electrodes in creating the advantages of monopolar RFA using a multiple-electrode switching system.

It has long been debated whether hepatic resection or RFA is the better treatment option for small HCCs (< 3 cm) in terms of survival and cost effectiveness [10]. This discrepancy regarding the best treatment strategy for small HCCs has mainly been related to the inability of complete local tumor control with RFA. According to a large retrospective study which reported the ten-year outcomes of percutaneous RFA as a first-line therapy of early HCC, the LTP rates were 27.0% and 36.9% at 5- and 10-year, respectively, for which the only significant risk factor was large tumor size (B = 0.584, *p* = 0.001) [42]. In fact, the development of LTP significantly shortened median recurrence-free survival and necessitated a higher number of interventional procedures [43]. Therefore, decreasing LTP after RFA has clinical importance in the management of HCC. In our study, the cumulative LTP and RFS rates of switching monopolar RFA using a separable clustered electrode at 3-year after RFA for small HCCs (mean tumor size of 1.8 cm) were 12.4% and 45.4%, respectively. In addition, switching monopolar RFA using a separable cluster electrode allowed the performance of the “no-tumor-touch” ablation technique or peripheral placement of electrodes in the index tumor. Considering that venous drainage changes from hepatic veins to sinusoid or peritumoral portal venules in progressed HCCs [44], placement of electrodes into the peritumoral portion or periphery of the index tumor can create more energy deposition in the periphery of the tumors and the peritumoral portion, which could induce thrombosis of the draining portal venules, which in turn may be advantageous in decreasing the risk of intraprocedural tumor cell seeding, especially in tumors located in subcapsular regions or perivascular areas [24]. Therefore, we expect that the enhanced effectiveness of local tumor control using the switching RFA system and a separable clustered electrode can contribute to enhancing the efficacy of RFA for the treatment of early stage HCCs.

Lastly, in our study, the technique success rate of RFA using a separable cluster electrode in the 98 tumors was 100%, and the major complication rate was 5%. Although the multiple electrode approach can cause more complexity in procedure planning, resulting in a relatively higher complication rate compared with that of conventional RFA using a single electrode [20, 34, 44], our study presented a comparable major complication rate compared to previous studies [20, 33, 45]. This may be attributed to the precise planning of the electrode path to the target tumor while avoiding the major vessels or bile ducts under the guidance of fusion imaging. Considering that it may be difficult for operators to visualize the segmental portal vein branches, hepatic arterial branches, bile ducts, and branches of the hepatic veins via US in patients with advanced liver cirrhosis who have a distorted anatomy and accentuated sonic attenuation, this capability of real-time fusion imaging allowing visualization of small vessels using contrast-enhanced CT or MR images and showing bile ducts using gadoxetic acid-enhanced MR images may be very useful in avoiding major complications related with electrodes.

There are a few limitations to our study. First, the LTP and RFS rates were retrospectively compared between the separable cluster electrode group and the multiple electrodes group. Although post hoc analyses revealed that there were no significant differences between the baseline characteristics of the two groups, the clinical outcomes need to be evaluated with prospective and well-controlled data. Second, the efficacy of a separable cluster electrode in ablating large HCCs was not sufficiently evaluated in this study. Theoretically, the combination of multiple separable cluster electrodes and a switching radiofrequency system should be useful in ablating a large volume at a given time. However, this study included HCCs < 5 cm, and the number of HCCs 3−5 cm was limited (n = 10).

In conclusion, multi-channel switching RFA using a separable cluster electrode under the guidance of a real-time fusion imaging system is a feasible and effective technique for the treatment of patients with small HCCs.

## Supporting Information

S1 TREND Checklist (ZIP)

S1 Protocol (DOCX)
