# Supplementary figures and images for: Switching Monopolar Radiofrequency Ablation Using a Separable Cluster Electrode in Patients with Hepatocellular Carcinoma: A Prospective Study
Source: PLoS One. 2016 Aug 30;11(8):e0161980. doi: 10.1371/journal.pone.0161980 (PMC5004876; doi:10.1371/journal.pone.0161980)

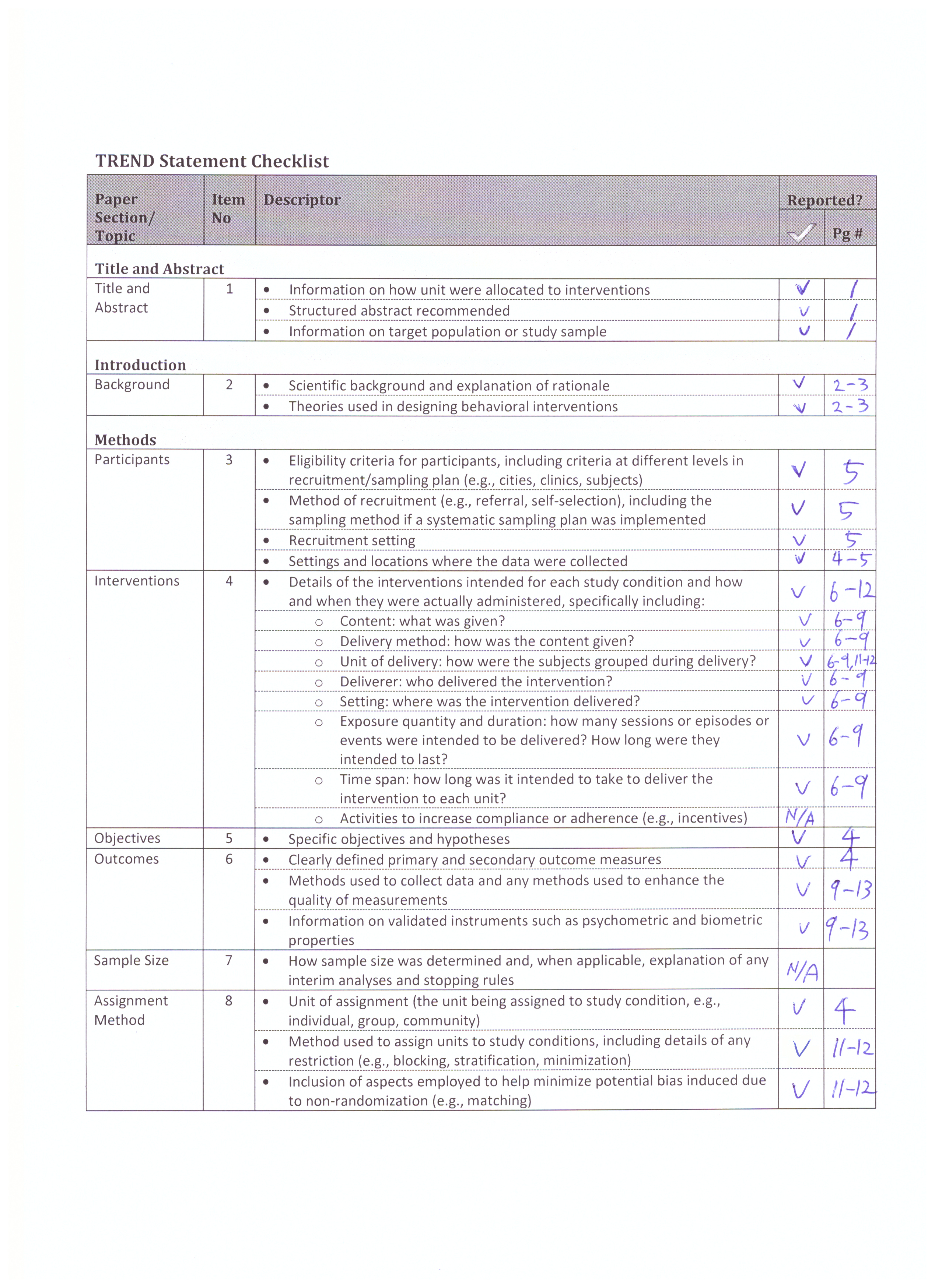

Supplement: S1 TREND Checklist — (ZIP) [file pone.0161980.s001.zip › S1_file_1.jpg]

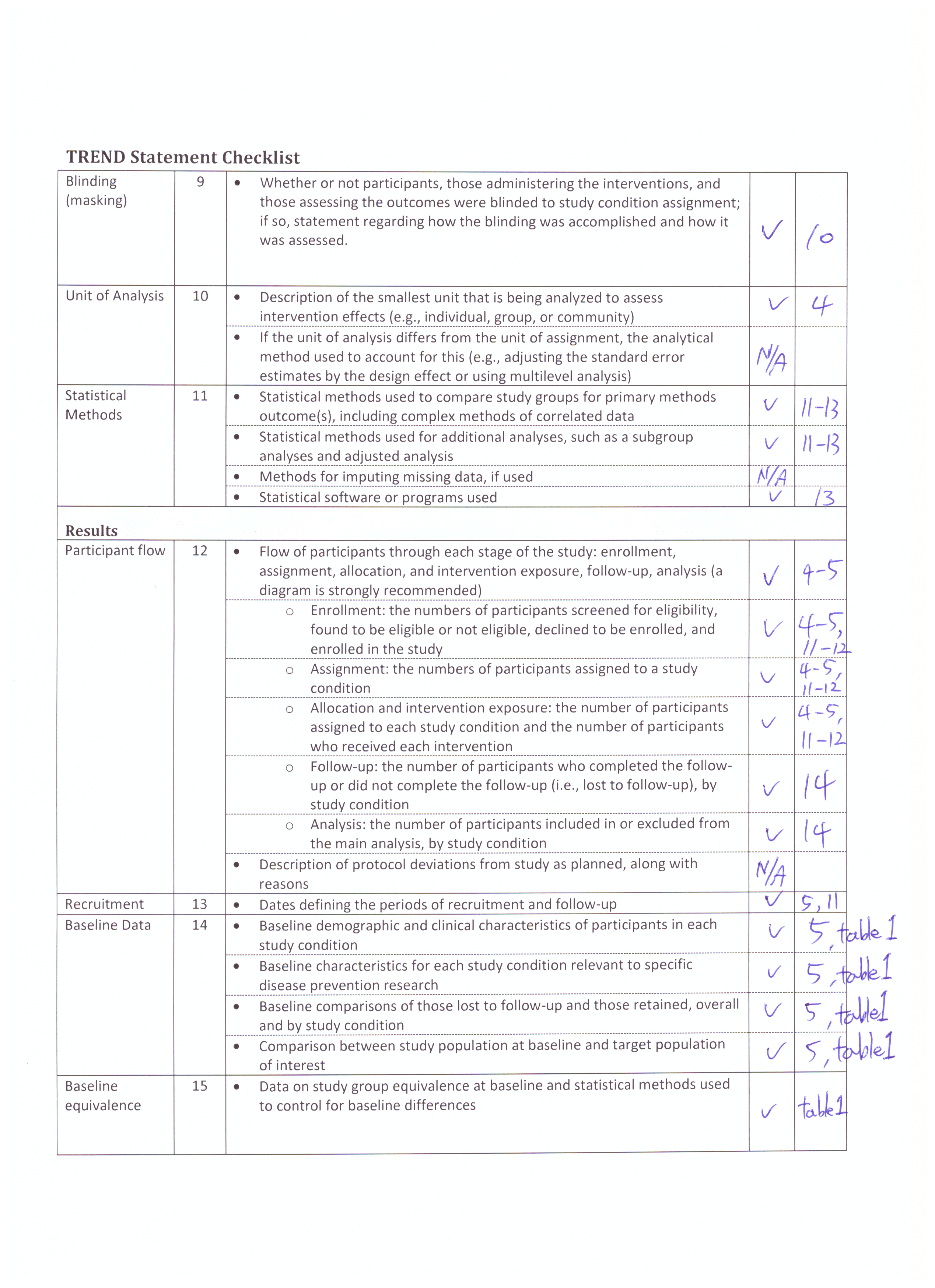

Supplement: S1 TREND Checklist — (ZIP) [file pone.0161980.s001.zip › S1_file_2.jpg]

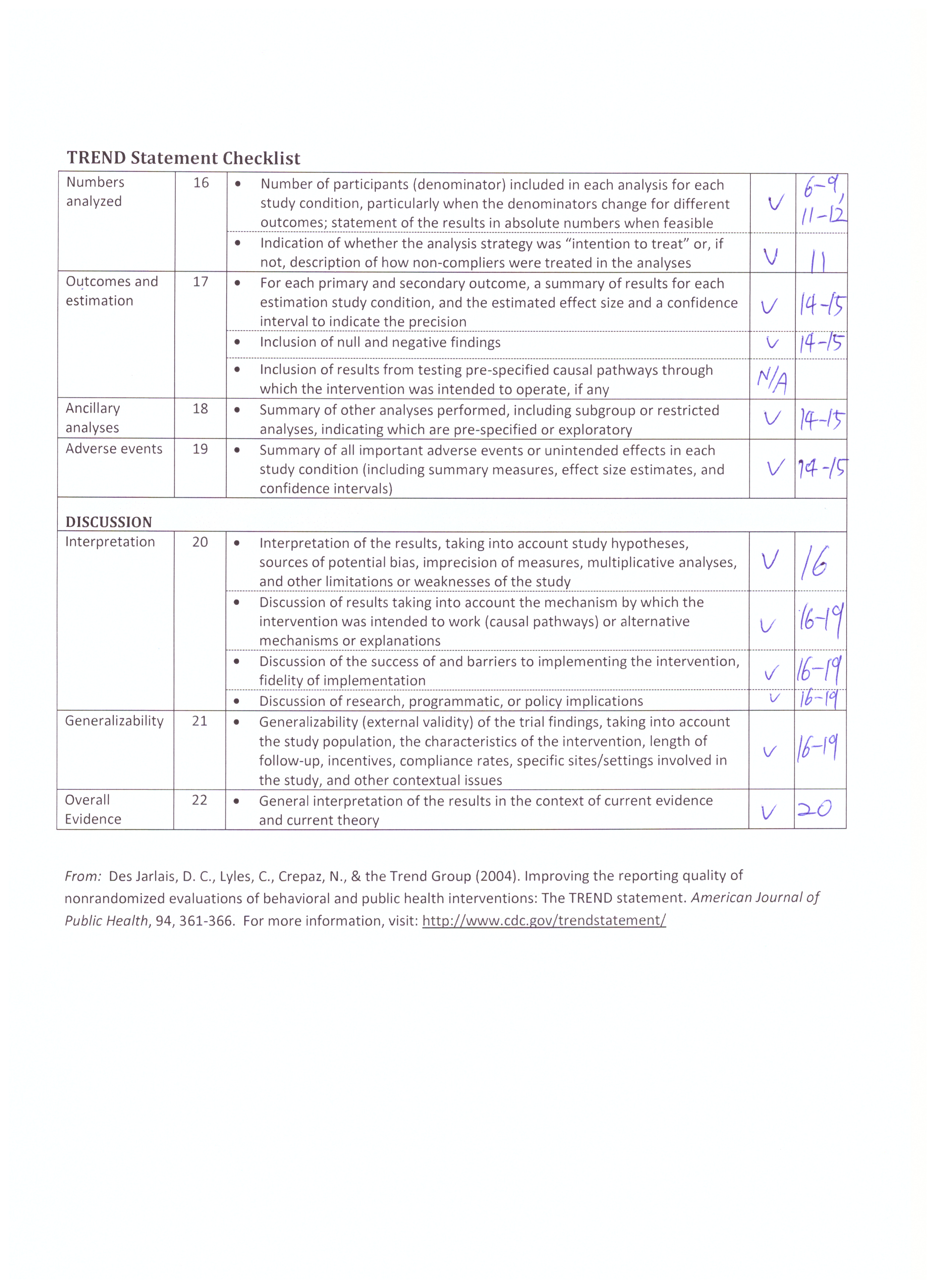

Supplement: S1 TREND Checklist — (ZIP) [file pone.0161980.s001.zip › S1_file_3.jpg]
