# Supplementary material for: Switching Monopolar Radiofrequency Ablation Using a Separable Cluster Electrode in Patients with Hepatocellular Carcinoma: A Prospective Study
Source: PLoS One. 2016 Aug 30;11(8):e0161980. doi: 10.1371/journal.pone.0161980 (PMC5004876; doi:10.1371/journal.pone.0161980)
Supplement: S1 Protocol — (DOCX) [file pone.0161980.s002.docx]

**Radiofrequency Ablation Using Octopus Electrodes for Treatment of Focal Liver Malignancies**

**Prospective, Single-center, Single-arm, Clinical Trial**

**Evaluating the Clinical Efficacy and Safety of a Novel Separable Cluster Electrode in Patients with Focal Liver Malignancies**

**Study protocol**

**Seoul National University Hospital**

**Department of Radiology**

**Primary Investigator: Jeong Min Lee, MD, PhD**

**Table of Contents**

1. Abstract ------------------------------------------------------------------------------------------------ 3

2. Background ------------------------------------------------------------------------------------------- 3

3. Objectives --------------------------------------------------------------------------------------------- 4

4. Endpoints ---------------------------------------------------------------------------------------------- 4

5. Eligible criteria ---------------------------------------------------------------------------------------- 5

6. Study design ------------------------------------------------------------------------------------------- 6

7. Required sample size -------------------------------------------------------------------------------- 6

8. Withdrawal criteria ---------------------------------------------------------------------------------- 6

9. General treatment guidelines ----------------------------------------------------------------------- 7

10. Follow-up -------------------------------------------------------------------------------------------- 9

11. Estimates --------------------------------------------------------------------------------------------- 9

12. Post-hoc subgroup analyses ---------------------------------------------------------------------- 10

13. Statistical analyses --------------------------------------------------------------------------------- 11

14. Ethical consideration ------------------------------------------------------------------------------ 12

15. References ------------------------------------------------------------------------------------------ 13

1. **Abstract**

This is a prospective, single-center, single-arm clinical study. The purpose of this study is to evaluate technical feasibility, safety, and clinical outcome of a novel separable cluster electrode for radiofrequency ablation (RFA) of hepatocellular carcinoma (HCC), in patients with focal liver malignancies

1. **Background**

According to the Barcelona Clinic Liver Cancer system, RFA is the only curative method among all non-surgical, locoregional treatment options in patients with HCC. RFA is also widely used as a curative treatment option for a variety of liver metastases, particularly colorectal liver metastases. Compared to surgery, RFA is less invasive, has less morbidity and requires shorter periods of hospitalization, yet provides comparable outcomes. Furthermore, the procedure is currently evolving as a competitor to surgery, especially in patients with single, small HCCs (< 3 cm in diameter). However, RFA is generally limited in creating a sufficiently large ablation volume for treating tumors larger than 2 cm in diameter with a sufficient tumor-free margin. Currently, various strategies are employed to create a sufficient ablation zone, including multi-tined or expandable electrodes to increase the surface area, internally cooled electrodes to diminish charring, perfusion electrodes to promote ionic availability, switching monopolar or multipolar controllers to provide a synergy of multiple applicators, and high-power generators to increase the power in order to overcome impedance. In this context, Lee et al recently reported that switching monopolar RFA using separable cluster electrodes (Octopus®, STARmed, Goyang, Korea), of which the inter-tine distances can be manipulated by the operator, was more efficient in creating a large ablation zone than using conventional cluster electrodes in an in vivo porcine study. This novel device may be more advantageous than conventional cluster electrodes by providing high flexibility to operators depending on the tumor size and shape, and by providing three times the active applicators with the same generator (i.e. a maximum of nine active tips are available with a generator containing three sockets). Although this separable cluster electrode has demonstrated promising results in a pre-clinical study, until now, the clinical feasibility and effectiveness of this novel device has yet to be demonstrated in human studies.

1. **Objectives**
   1. To evaluate the clinical feasibility and outcomes of switching monopolar RFA using a separable cluster electrode in patients with HCC.
   2. To compare the therapeutic outcomes and safety of the study patients with those of a matched historical control group
2. **Endpoints**
   1. Primary Outcome Measures:
      1. Major complication rate after RFA [ Time Frame: 30 days after RFA ]
      2. Technical success [ Time Frame: 1 day after RFA ]
   2. Secondary Outcome Measures:
      1. Local control (tumor progression) rate [ Time Frame: 12 months after RFA ]
      2. Recurrence-free survival rate [ Time Frame: 24 months after RFA ]
   3. Other Outcome Measures:
      1. Total procedure time [ Time Frame: 1 day after RFA ]
      2. Ablative zone volume [ Time Frame: 1 day after RFA ]
3. **Eligible criteria**
   1. Inclusion Criteria:
      1. HCC diagnosed on biopsy, OR typical imaging features of HCC on CT or MRI according to AASLD guideline
      2. 1-3 HCCs equal to or smaller than 5 cm in the liver
      3. no direct contact with or invasion into the hepatic hilar structures or inferior vena cava
      4. treatment-naïve HCC
      5. Eastern Cooperative Oncology Group performance status of 0
   2. Exclusion Criteria: Patients with any of followings are excluded.
      1. Patients with uncontrolled coagulopathy
      2. Patients with Child-Pugh classification C
      3. Patients with tumor invasion into the portal vein or hepatic vein
      4. Extrahepatic spread
4. **Study design**
   1. Prospective, single-center, single-arm clinical trials
   2. Phase II clinical trials for novel separable cluster electrode which demonstrated potential efficacy and feasibility on pre-clinical study.
5. **Required sample size**
   1. 196 patients with focal liver malignancies
6. **Withdrawal criteria**
   1. All participants have the right to withdraw at any point during the study without risk of future prejudice. Any investigators or medical staffs may discontinue the study procedure(s) in any participant at any time if medically necessary.
   2. If the study procedures are discontinued for any participant, the reason should be recorded, and the primary investigator must be notified promptly. Participants will not be replaced in this study.
7. **General treatment guidelines**
   1. CT Image Acquisition for Image Fusion
      1. Monophasic late arterial phase CT scans on the same day as RFA (Discovery CT750HD; GE Healthcare, Waukesha, WI, USA).
      2. Intravenous administration of a nonionic iodinated contrast agent (1 mL/kg of iopromide, Ultravist 370; Bayer Healthcare, Berlin, Germany) at a rate of 2-4 mL/sec.
   2. RFA
      1. Ablation Protocol
         1. The goal of RFA is to achieve complete ablation of both the index tumor and an ablation margin (0.5 to 1.0 cm) in the normal liver parenchyma.
         2. Conscious sedation, with close monitoring of the vital signs.
         3. Local anesthesia using a subcutaneous injection of 5−15 mL of 1% lidocaine (Dai Han Pharm, Seoul, Korea).
         4. One cycle of ablation for approximately 10 minutes in tumors < 2.5 cm and for 18 minutes in tumors > 2.5 cm.
         5. In cases of incomplete ablation, additional cycle(s) of RFA were done followed by repositioning of the electrode(s), as appropriate.
         6. Active tip adjustment among three options, 2.5 cm, 3.0 cm, and 4.0 cm, depending on the tumor size, location, etc. In general, if the tumor was smaller than 1.5 cm in its longest axis, electrodes with 2.5 cm active tips were used using the “no-tumor-touch” technique [32].
         7. Grounding by attaching four, dispersive pads to the patients’ thighs.
         8. Artificial ascites (5% dextrose solution) for subcapsular tumors or tumors located near the diaphragm
         9. Tract ablation after tumor ablation, by maintaining the active tips at 90 ºC while retracting the electrodes.
      2. Fusion Imaging-guidance
         1. A real time fusion imaging technique between pre-procedural CT imaging and intra-procedural US imaging (PercuNAV; Philips Healthcare, Best, Netherlands).
         2. Conscious sedation of the patients, during image registration for fusion.
         3. (Optional) Artificial ascites instillation before image fusion.
         4. Impedance-switching algorithm to deliver energy
      3. Switching radiofrequency system with a Separable Cluster Electrode
         1. Separable cluster electrode (Octopus®; STARmed), in which the inter-tine distances can be manipulated by the operator.
         2. A 200-watt, multichannel, radiofrequency system (Viva RF System; STARmed) with three independently adjustable generators
         3. Switching monopolar RF energy delivery mode; the active electrode will be switched when the impedance raises 50 Ω above the baseline or when the ablation time passes 30-second.
         4. Circulating chilled normal saline in the lumen of the electrodes.
         5. Other detailed ablation algorithm of energy application according to the manufacturer’s instructions.
   3. Post-RFA imaging
      1. Two phase (arterial, portal) CT imaging with multiplanar reconstruction (axial, coronal, and sagittal) after the RFA.
      2. Intravenous administration of contrast medium (1.35 mL/kg of Ultravist 370; Bayer Healthcare) at a rate of 2.0 to 4.0 mL/s using a power injector.
8. **Follow-up**
   1. initial follow-up one month after the procedure with quadriphasic (unenhanced, arterial, portal, and delayed phase) CT imaging
   2. Subsequent follow-up every three months with quadriphasic (unenhanced, arterial, portal, and delayed phase) CT imaging
9. **Estimates**
   1. All images were analyzed by a blinded radiologist.
   2. Technique success: complete coverage of an index tumor and an extended ablation zone beyond the tumor border on immediate post-RFA CT, according to the standardized terminology of the International Working Group on Image-Guided Tumor Ablation.
   3. Ablation volume = (π × Dmax ×Dmin ×Dvert) / 6

, where Dmax and Dmin are the longest and shortest diameters of the ablation zone (axial image), and Dvert is the longest vertical diameter (coronal) image.

- 1. Total procedure time: the time between the start of planning ultrasonography until the awakening of the patient from conscious sedation
  2. Ablation time: the time when energy was actively delivered via the electrodes
  3. Major complication: events increasing the level of care or lengthening the hospital stay.
  4. Procedure-related death: death within 30 days after the RFA
  5. Post-ablation syndrome: transient and self-limiting symptoms of low-grade fever and/or general malaise.
  6. Technique efficacy: complete ablation of the index tumor on 1 month follow-up CT images
  7. Local tumor progression: tumor foci appeared at the edge of the ablation zone.
  8. Recurrence-free survival: time after RFA during death or the first recurrence of the HCC on follow-up imaging

1. **Post-hoc subgroup analyses**
   1. Purpose: to obtain sufficient statistical power (longer follow-up period) and objectively compare the outcomes of separable cluster electrode with that of routine practice with similar manners.
   2. Endpoints
      1. Primary endpoint: cumulative 1-year, 2-year, and 3-year local tumor progression rates
      2. Secondary endpoint: cumulative 1-year, 2-year, and 3-year recurrence-free survival rates
      3. Tertiary endpoints: technique success rate and major complication rate
   3. End of patients follow-up: July 2015 (2-year after the last patient’s enrollment)
   4. Subgroup: patients with Treatment-naïve HCC (79 patients with 98 HCCs)
   5. Matched historical comparison group (74 patients with 88 HCCs).
      1. From January 2011 to July 2013
      2. Patient who satisfied the same inclusion/exclusion criteria
      3. The same RFA protocols and devices but with multiple internally-cooled electrodes, instead of separable cluster electrodes
2. **Statistical analyses**
   1. Descriptive statistics: technique success rate, ablation volume, total procedure time, ablation time major complication rate, technique efficacy
   2. Survival analyses: time to local tumor progression, recurrence-free survival
      1. Kaplan-Meier curve and log-rank test
      2. Univariate and multivariate Cox proportional hazard regression for potential factors affecting the survival estimates.

1. **Ethical considerations**
   1. Early stopping rule
      1. Major complications are classified according to Common Terminology Criteria for Adverse Event (CTCAE) version 4.0.
      2. If two or more unexpected adverse events with a severity of CTCAE Grade 4 (life-threatening) or 5 (death) are reported, then the Institutional Review Board (IRB) will urgently investigate whether the events are attributable to the study procedures
      3. Depending on the urgent investigation, the IRB can recommend early termination of this study
   2. Potential conflict of interest
      1. Financial support and the investigational RF ablation device for our study were provided by STARmed (Goyang, Korea): grant ID #1441
      2. The authors had complete control of the data and information submitted for publication, which was unbiased by industry.
   3. Ethics and responsibility
      1. This study will be conducted in accordance with the IRB-approved study protocol, the institutional standard operating procedures, the institutional IRB regulations, the International Conference on Harmonisation (ICH) Good Clinical Practice (GCP) guidelines, the Declaration of Helsinki, and applicable government regulations.
      2. The study protocol, Informed Consent Form, and any amendments of them will be submitted to the institutional IRB for formal approval of the study conduct.
   4. Informed consent form
      1. All participants will be given an IRB-approved Informed Consent Form providing sufficient information for participants to make informed decisions about their participation in this study.
      2. The Informed Consent Form must be signed and dated by the participant or a legally acceptable representative and the primary investigator-designated research staff before the participant is subjected to any study procedures..
2. **References**
   1. Bruix J, Sherman M, American Association for the Study of Liver Diseases (2011) Management of hepatocellular carcinoma: an update. Hepatology 53: 1020-1022.
   2. European Association for the Study of the Liver, European Organisation for Reresarch and Treatment of Cancer (2012) EASL-EORTC clinical practice guidelines: management of hepatocellular carcinoma. J Hepatol 56 :908-943.
   3. Woo S, Lee JM, Yoon JH, Joo I, Kim SH, Lee JY, et al. (2013) Small- and medium-sized hepatocellular carcinomas: monopolar radiofrequency ablation with a multiple-electrode switching system-mid-term results. Radiology 268: 589-600.
   4. Lee ES, Lee JM, Kim WS, Choi SH, Joo I, Kim M, et al. (2012) Multiple-electrode radiofrequency ablations using Octopus(R) electrodes in an in vivo porcine liver model. Br J Radiol 85:e609-e615.
   5. Ahmed M, Solbiati L, Brace CL, Breen DJ, Callstrom MR, Charboneau JW, et al. (2014) Image-guided tumor ablation: standardization of terminology and reporting criteria--a 10-year update. Radiology 273: 241-260.
   6. Yoon JH, Lee JM, Hwang EJ, Hwang IP, Baek J, Han JK, et al. (2014) Monopolar radiofrequency ablation using a dual-switching system and a separable clustered electrode: evaluation of the in vivo efficiency. Korean J Radiol 15: 235-244.
   7. Yoon JH, Lee JM, Han JK, Choi BI (2013) Dual switching monopolar radiofrequency ablation using a separable clustered electrode: comparison with consecutive and switching monopolar modes in ex vivo bovine livers. Korean J Radiol 14: 403-411.
   8. Lee DH, Lee JM, Lee JY, Kim SH, Yoon JH, Kim YJ, et al. (2014) Radiofrequency ablation of hepatocellular carcinoma as first-line treatment: long-term results and prognostic factors in 162 patients with cirrhosis. Radiology 270: 900-909.
